# The glassy random laser: replica symmetry breaking in the intensity fluctuations of emission spectra

**DOI:** 10.1038/srep16792

**Published:** 2015-11-30

**Authors:** Fabrizio Antenucci, Andrea Crisanti, Luca Leuzzi

**Affiliations:** 1 NANOTEC-CNR, Institute of Nanotechnology, Soft and Living Matter Laboratory, Rome, Piazzale A. Moro 2, I-00185, Roma, Italy; 2 Dipartimento di Fisica, Università di Roma “Sapienza”, Piazzale A. Moro 2, I-00185, Roma, Italy; 3 ISC-CNR, UOS Sapienza, Piazzale A. Moro 2, I-00185, Roma, Italy

## Abstract

The behavior of a newly introduced overlap parameter, measuring the correlation between intensity fluctuations of waves in random media, is analyzed in different physical regimes, with varying amount of disorder and non-linearity. This order parameter allows to identify the laser transition in random media and describes its possible glassy nature in terms of emission spectra data, the only data so far accessible in random laser measurements. The theoretical analysis is performed in terms of the complex spherical spin-glass model, a statistical mechanical model describing the onset and the behavior of random lasers in open cavities. Replica Symmetry Breaking theory allows to discern different kinds of randomness in the high pumping regime, including the most complex and intriguing glassy randomness. The outcome of the theoretical study is, eventually, compared to recent intensity fluctuation overlap measurements demonstrating the validity of the theory and providing a straightforward interpretation of qualitatively different spectral behaviors in different random lasers.

Light amplification and propagation through random media have attracted much attention in recent years, with present-day applications to, e.g., speckle-free imaging and biomedical diagnostics^1^, chip-based spectrometers^2^,^3^,^4^, laser paints^5^ and cryptography^6^. Whatever the amplifying medium, ordered or random, in a closed or in an open cavity, two are the basic ingredients to produce laser in any optically active system: *amplification* and *feedback*. In closed cavities the electromagnetic modes straightforwardly depend on the cavity geometry. In cavity-less random media, instead, some kind of modes are established by spontaneous emission and are localized in closed photonic trajectories by means of multiple scattering. Indeed, the phenomenon of *amplified spontaneous emission* (ASE) can occur even in systems without any optical cavity, whose fluorescence spectrum is simply determined by the gain curve of the active medium^7^,^8^,^9^,^10^,^11^,^12^. When, because of an external pumping, the multiple-scattering feedback process becomes strong, amplification by *stimulated* emission is established in the random medium and we have a *Random Laser* (RL)^13^. The feedback is, here, associated to the existence of well-defined long-lived modes, characterized by a definite frequency and a spatial pattern of the electromagnetic field inside the material. Modes are expressed as *slow amplitude* contributions to the electromagnetic field expansion in terms of spatial mode eigenvectors 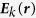:


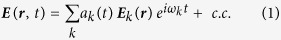


The complex amplitudes *a*_*k*_(*t*) of these slow modes turn out to be the fundamental degree of freedom in the statistical mechanical modeling of interacting modes^14^,^15^, while the irregularity of their spatial profiles results into *quenched* disordered mode interactions. By quenched we mean that the interaction strengths are time independent^16^, as it occurs, in practice, when they change on time-scales much longer then the typical amplification time-scales, longer than the RL lifetime itself and possibly reproducible in a series of apart RL measurements on the same sample under the same experimental conditions.

At least in some random media, the RL action presents the peculiar property of displaying strong non-trivial spectral fluctuations^17^,^18^,^19^,^20^,^21^,^22^ from one excitation pulse of the pumping laser to another one^23^,^24^,^25^,^26^. These will be termed *shot-to-shot* fluctuations from now on. If in spectral fluctuation measurements the scattering particles and all external experimental conditions are kept constant, these fluctuations will only be due to the initial configuration of pre-pumping electromagnetic modes occurring because of spontaneous emissions.

A connection to statistical mechanical models with quenched disordered interaction, i.e., spin-glass models^16^,^27^,^28^,^29^, has been recently established^14^,^15^,^30^,^31^,^32^,^33^, providing a new point of view on the shot-to-shot fluctuations phenomenon. The leading mechanism for the non-deterministic activation of the modes is here identified with the *frustration* induced by the disordered interactions^27^ and the consequent presence of a large number of equivalent *states*. For *state* we mean a given ensemble of activated mode configurations, specified by their own wavelengths, phases and intensities, realized by very many emissions on time-scales of the order of the duration of the shot, that is, of the RL life-time itself. The diverse spectral realizations are, thus, conjectured to correspond to a glassy behavior consisting in many equivalent degenerate states constituting the RL regime. This glassy light regime is associated to an effective thermodynamic phase where the tendency of the modes to oscillate coherently in intensity is frustrated: in the language of the replica theory^27^, it corresponds to a phase where the symmetry among equivalent replicas is spontaneously broken^34^ and the overlap (i.e., the similarity) between the configurations of the mode amplitudes display a nontrivial structure^15^,^35^. Identical copies of the system show different sets of amplitude equilibrium configurations, as the ergodicity is broken in many distinct states^36^.

From an experimental point of view, the direct evaluation of the overlap between complex amplitudes and its probability distribution, i.e., the standard order parameter of the theory, requires the measure of the mode phases in the coherent regime. Such measure is not available so far, because of the low intensity of the RL emission (with respect to standard cavity lasers). The lack of a direct experimental knowledge of the whole overlap probability distribution is common, as well, to the original prototype systems for which replica symmetry breaking (RSB) theory was first developed, i.e., spin-glasses^37^,^38^, and also to structural glasses, one of the fields of major application of the theory^39^,^40^,^41^,^42^,^43^.

An experimental validation of such *random-glassy* laser connection, and, particularly, of the RSB predicted by the theory, has, nevertheless, recently been put forward in ref. 44, measuring the overlap between *intensity fluctuations* of the spectral emission. In the present work, we adopt a general model for cavity-less random lasers, in which not only the mode phases^30^,^31^,^32^,^33^ but the whole complex amplitudes are considered as the fundamental degrees of freedom of the problem^14^,^15^. In this framework we are able to demonstrate that RSB occurring in the standard amplitude overlap can, in principle, be observed in the intensity fluctuation overlap (actually a coarse-graining of the former) and vice versa. This development provides a theoretical setting to explain existing experimental results and to motivate similar measurements in diverse RL systems. Our approach also clarifies why RSB is found only in RL’s in which mode couplings can be considered fixed (termed *quenched*) for all shots. In liquid compounds, instead, as a TiO_2_ dispersion in Rhodamine B-ethylene glycol solution, no evidence for RSB is found^44^.

## The Complex Amplitude Model

The statistical approach we adopt is based on the hypothesis of effective equilibrium. The non-equilibrium steady-state of a laser can be described as an effective equilibrium state at an effective temperature linked to the pumping rate of the source and to the true environment temperature (associated, e.g., to the noise of the spontaneous emission). In order to implement such description, the gain behavior is chosen in such a way to guarantee that the global optical power 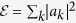 remains constant. As a consequence, in the mean-field limit, the complex amplitudes 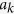 statistics is described by the general Hamiltonian^14^,^15^


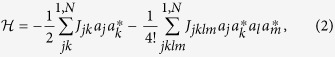


where the sums are unrestricted and 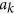 are *N* complex amplitude variables subject to the global power constraint 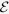  = const. The coupling strengths are here quenched independent random variables with mean 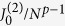 and variance 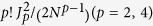, whose scaling with 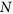 guarantees an extensive Hamiltonian and thermodynamic convergence. For large 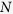, the corresponding probability distribution can be taken Gaussian without loss of generality. Let us also define the degree of disorder 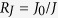 and the pumping rate 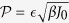 with 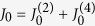 and 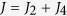, and where *β* is the inverse of the environment temperature.

This model can be derived in a multimode laser theory for open and irregular random resonators^15^. The openness of the cavity can be encoded into the definition of the electromagnetic modes using, e.g., the system-and-bath approach of ref. 45, in which the contributions of radiative and localized modes are separated by Feshbach projection^46^ onto two orthogonal subspaces. This leads to an effective theory on the subspace of localized modes in which they exchange a linear off-diagonal effective damping coupling^45^,^47^,^48^. In terms of the interaction parameters, we also define the strength of the openness as the inverse strength of the nonlinear interaction coupling with respect to the off-diagonal linear coupling 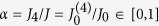. In a closed cavity the linear dumping is absent and it corresponds to *α* = 1.

In a standard semiclassical approach, the field is expressed in the slow amplitude basis, equation (1), where each mode displays a determined frequency. The lifetimes of these modes are assumed to be much longer than the characteristic times of population inversion and amplification processes, so that the atomic variables can be adiabatically removed and result in an effective interaction between the electromagnetic modes. The nonlinear couplings are, indeed, nonzero only for the terms 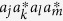 that meet the *frequency matching condition*^49^,^50^,^51^,


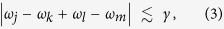



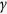
 being the finite linewidth of the modes.

The mean-field approximation of the model equation (2) is exact when the probability distribution of the couplings is the same for all the mode couples (*j, k*) and tetrads (*j, k, l, m*). This is true, e.g., when mode extensions scale with the volume occupied by the active medium and their spectrum has a narrow-bandwidth around some given central frequency *ω*_0_, i.e., 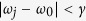, 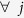, so that the frequency matching condition, cf. equation (3), always holds.

## Results

### The Random Laser Transition

Given the quenched randomness of the 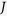
’s, any observable depends on the particular realization of the disorder. Thus, the relevant quantity is the disorder averaged free energy 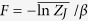, where the overline denotes the average over the distribution of quenched disordered couplings. This can be analytically evaluated using the replica method^27^,^37^, as reported in the Methods. In this procedure the evaluation of the relevant thermodynamic quantities is achieved considering 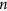 identical replicas (i.e., copies) of the system that act as probes exploring the multi-state phase space of the system. Further on, evaluating the distance between the replicas in terms of their similarity, termed *overlap*, one can retrieve the physical overlaps of the thermodynamic states^35^.

In the complex amplitude spherical model, equation (2), the order parameter of the replica theory turns out to be given by the overlap between amplitudes of replica a and replica b:


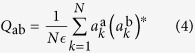


where 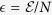. This overlap 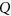 identifies the onset of a RL regime at a given critical value of the pumping, or, otherwise, at a critical temperature at fixed pumping. We notice that the latter behavior is in qualitative agreement with the experimental results of refs 52, 53, 54 where random lasing appears to occur decreasing temperature, besides increasing pumping.

Any nontrivial structure of the values taken by *Q*_ab_ implies that identical copies of the system, with the same interaction network and submitted to the same thermodynamic conditions, show different sets of values for microscopic observables at equilibrium and the ergodicity is broken in distinct equivalent states.

To our knowledge, from an experimental point of view, no phase correlation measurements, required for the evaluation of the complex amplitudes 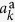 and, consequently, of 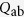, is available so far in random media. Only magnitudes 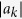 are measured and not their phases 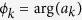. The experimental reconstruction of the distribution of the values of equation (4) is, thus, unfeasible.

### Real Replicas

In recent experiments^44^, shot-to-shot fluctuations of intensity spectra in an amorphous solid RL, a functionalized thiophene-based oligomer named thienyl-S,S-dioxide quinquethiophene (T5COx), are measured and analyzed. Since the sample remains under identical experimental conditions shot after shot, 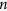 different shots of RL emission correspond to 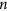 *real replicas* and one can measure the overlap between *intensity fluctuations* of two real replicas. In these experiments, the set of the activated modes emitting after the shot 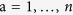, whose available coarse-grained degree of freedom is the intensity 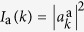, is observed to change from shot to shot.

When, during a single shot of the pumping source, the number of stimulated emission processes taking place is very large, the configurations of the mode dynamics can be considered as pertaining to a thermodynamic state. In terms of the *photonic bomb* language of Letohkov^10^, e.g., this is a situation in which the typical amplification time is much shorter than the photons lifetime inside the medium, i.e., of the lifetime of stochastic resonators supporting the localized optical modes. The possible observation of numerous different states from shot to shot is, consequently, an evidence of a thermodynamic phase described by a corrugated free energy landscape composed of many valleys separated by barriers.

### Intensity Fluctuation Overlap (IFO)

Having as only experimentally available degree of freedom the intensities, one defines a suitable overlap based on their acquisition in different shots. To this aim, one first determines the average emission spectrum 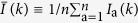. Then, terming 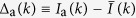 the intensity fluctuation of shot a around the average profile, one can define the overlap between the normalized intensity fluctuation of shots a and b as^44^:


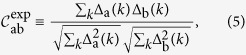


where the index 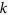 denotes now the frequency, i.e., the experimental accessible equivalent of a mode index, depending on the spectral resolution. The overlap is measured between the fluctuations of intensity, rather than the straight intensities, to exclude the effects due to the amplified spontaneous emission. From 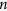 measured spectra one can calculate the 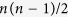 values of the IFO 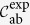 and its distribution 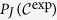. Its average 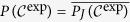 can be computed by repeated spectral measurements acquired on different samples. By different samples we, actually, mean different realizations of the microscopic disordered realization of scatterers positions as faced by the incoming pumping light beam. More precisely, one can realize a different realization by turning the material sample or, if the beam section is smaller than the random medium, by illuminating a different region with the pump laser spot.

If the variations of the normalization factors 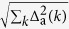 in Eq. (5) are neglected with respect to fluctuations 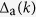, in the 2 + 4 complex amplitude spin-glass model given by equation (2), the matrix


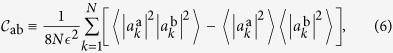


is the model equivalent of the IFO, up to an overall sign. Indeed, equation (6), defined in the dominion [0, 1], holds with the prescription that 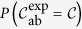 corresponds to 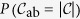. To compare with experimental results we will use symmetrized 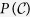, 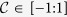, without any loss of generality.

### IFO vs. standard overlap relationship

The average in equation (6) can be carried out using the replicated action derived in the Methods, cf. equation (14). This leads to the following relationship between the IFO and the standard order parameters:


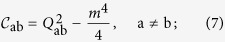


where 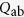 is defined in equation (4), and


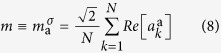


is the parameter of global coherence (cf. Methods). According to equation (7), if a RSB occurs in the standard overlap 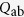 it propagates with the same structure to the IFO 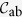. We, thus, have a theoretical well funded tool to detect RSB in experimental data. We stress that this analysis could have not been possible in 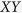 models with quenched amplitudes considered in previous works^30^,^31^,^32^ because there the intensities of the modes are kept fixed during the mode dynamics.

**Figure 1:**
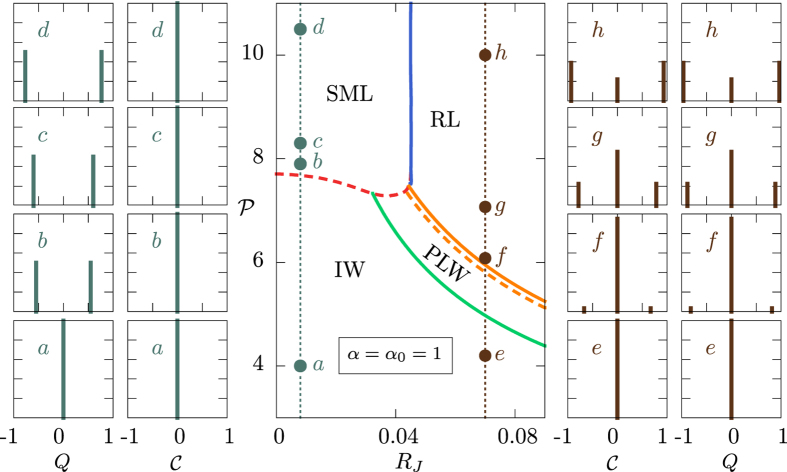
Laser transition triptych in a closed cavity for varying disorder. In the central panel the phase diagram 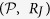 is displayed for a closed cavity (nonlinearity strength *α* = 1) in terms of the four possible optical regimes^14^,^15^: incoherent wave (IW), standard mode locking (SML), phase locking wave (PLW) and random laser (RL). Two pumping paths across the lasing thresholds are shown as dotted lines, at 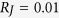 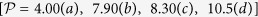 and 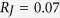 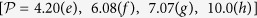. In the left panels 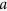 to 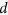 the behavior the distributions of IFO, 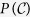, and standard overlap, 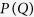, across the ordered ML laser threshold are reported. The transition is discontinuous in the standard Parisi distribution 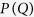, whereas 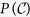 is invariant. In the right panels 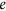 to 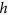 the IFO and standard overlap distributions are shown for the RL transition: as 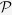 increases, we show that the low 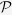 solution is replica symmetric (*e*), while above threshold it becomes discontinuously 1RSB (*f, g, h*).

**Figure 2:**
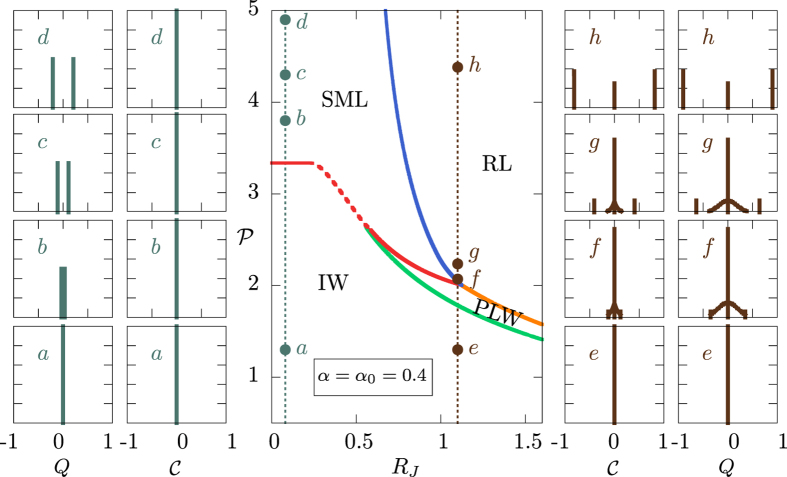
Laser transition triptych in an open cavity for varying disorder. In the central panel the phase diagram 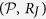 is displayed for an open cavity (nonlinearity strength *α* = 0.4) in terms of the four possible optical regimes^14^,^15^: incoherent wave (IW), standard mode locking (SML), phase locking wave (PLW) and random laser (RL). Two pumping paths across the lasing tresholds are shown as dotted lines, at 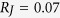 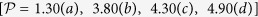 and 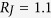 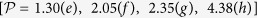. In the left panels 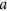 to 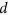 the behavior of IFO and standard overlap distributions across the ordered ML laser threshold are reported. The transition is now continuous in the order parameters 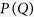, while 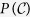 does not change below and above threshold. In the right panels 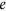 to 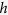 the IFO and standard overlap distributions are shown for the RL transition. As 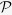 increases we show that the low optical power solution is replica symmetric (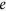), soon above threshold the solution is FRSB (*f*), further increasing 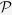 the solution becomes 1 + FRSB (*g*) and, eventually, for large pumping it is 1RSB (*h*). The transition is continuous in the order parameters 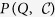.

In equation (7) we have considered the most general case in which a high pumping regime can display both a global coherence (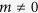) and a multi-state non-trivial structure for the amplitude configurations (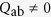). This mixing physically occurs for a degree of disorder 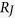 next to the tolerance value beyond which standard mode locking (SML) breaks down, leaving place to glassy random lasing. This is displayed in the phase diagrams in the central panels of the triptych Figs 1 and 2, as the boundary lines between SML (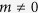) and glassy random laser (*m* = 0 but 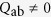) at large 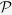.

The low pumping regime is replica symmetric for any 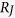, with *m* = 0 and *Q*_ab_ = 0 for 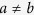
14, implying a Dirac delta probability 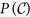, peaked in zero, both in the incoherent wave (IW) and in the phase locking wave (PLW) in Figs 1 and 2 (cf. also Methods).

### Replica Symmetric standard mode-locking laser

For weak disorder at high pumping 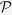, for every 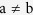 the relationship 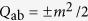, with 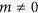, holds between the overlap and the (replica independent) global coherence parameter. In other words, the high pumping laser regime is replica symmetric, as well, and the 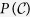 is a Dirac delta function in zero, once again. The laser regime for negligible disorder (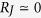) corresponds to a standard mode-locking laser in an ordered cavity^49^,^50^ with spectral resonances equispaced in wavelength and smoothly distributed in intensity. For weak, though not very small, disorder, as, e.g., in the open cavity phase diagram of Fig. 2 (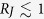), the laser regime could also correspond to a deterministic (i.e., non-glassy) random laser, with spectral resonances indeed at random wavelengths with random intensities, yet always with the same pattern for each experiment on the same sample under the same external conditions. From a classical statistical mechanics point of view these two cases are equivalent (in terms of the order parameters equations (4,8)) and they are referred simply as SML in the phase diagrams. We will come back to this kind of random lasers when discussing known experimental realizations.

Remarkably, though in terms of the parameter 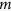 the standard mode locked regime is clearly different from the fluorescence regime, and so is 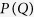, cf. left panels 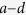 of Figs 1 and 2 and refs 14,15, *the IFO distribution does not change below and above the standard mode-locking transition*. This has been observed in preliminary measurements on a Q-switched pulsed Nd-Yag standard laser in ref. 44. Indeed, the overlap of the model in equation (6) is between local fluctuations of intensity on different replicas, so a global ordering is invariably taken away (cf. Methods).

### Onset of RSB across the random laser transition

For strong disorder the distribution of the coupling 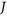
’s yields a non-negligible amount of both positive and negative values, inducing frustration in the modes interaction. Take, for instance and simplicity, three complex amplitudes 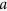, pairwise interacting on a triangle. Each of the three bonds connecting the three modes can randomly acquire positive or negative values. Modes connected by positive bonds will tend to align (in the complex plane), whereas modes connected by negative bonds will tend to counter-align. If, e.g., in the triangle two bonds are positive and one negative, no single configuration of modes alignments will satisfy all interactions, minimizing the Hamiltonian. The system will then settle into one of different degenerate configurations with the lowest realizable energy. Frustration is, thus, the impossibility of finding a unique way of satisfying all bonds. Frustration is a necessary condition for the onset of glassiness. When the pumping increases above threshold, 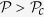, the replica symmetry is broken and the distribution of 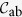 becomes nontrivial, cf. panels *f, g, h* in Figs 1 and 2.

### Phase Diagrams and Overlap Distributions

Several scenarios are possible at the lasing transitions, exemplified in the paradigmatic cases of Figs 1 and 2. In Fig. 1 we show the phase diagram and the behavior of the overlap distribution 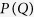 and its relative symmetrized IFO distribution 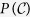 in a closed cavity (*α* = 1) where linear dumping is absent. In Fig. 2 the behavior of 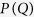 and 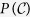 is shown across the laser threshold in an open cavity (*α* = 0.4 < 1), where the linear dumping is competing with non-linearity. In both the closed and the open cavity scenarios we illustrate two different critical regimes: the onset of standard mode locking at low disorder and the transition to random lasing for large 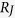.

In a closed cavity situation, *α* = 1, 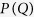 is discontinuous at the standard mode-locking laser transition, while 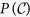 is unaffected. In this case the transition itself is discontinuous in the thermodynamic sense: the internal energy^14^,^50^ and the coherence parameter 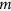 (or the overlap 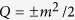) are discontinuous, see left panel of the closed cavity triptych in Fig. 1. Here the distribution 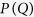 has two values trivially linked to the two possible values of the nonzero parameter, 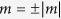. At the RL transition (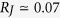), alternatively, the 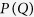, and similarly 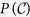, changes in a nontrivial way: two different values, a zero and a nonzero one, are possible as the pumping is increased. In this situation the RL regime is one step RSB (1RSB) and the transition is a so-called *random* first order (RFOT) in glassy physics terming^16^. In the RFOT scenario the static (ideal) glass transition is preceded by a glassy dynamic arrest (drawn as a dashed line in the central panel of Fig. 1)^14^,^15^: a photonic system in this case should show the typical two-step dynamical relaxation for the time correlation function of light modes, in the same universality class of the mode-coupling theory for structural glasses. In this kind of transition there is no latent heat^15^, yet a new value for the overlap discontinuously appears at the transition, cf. right panel of Fig. 1.

In the cavity-less scenario *α* = 0.4, instead, 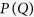 is continuous at the ordered ML transition. Indeed, a nonzero value for 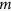 increases continuously from zero as it can be observed looking at the peaks of 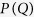 in panels *a* and *b* of Fig. 2, where 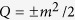. As in the closed cavity scenario, the 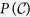 of the SML does not change across the threshold. At the onset of the RL regime, illustrated in Fig. 2 for 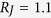, the change in 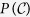 is rather meaningful. At and just above the threshold, 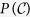 displays a continuous part between the central peak in 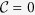 and the two side peaks, as displayed in panel 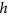 of Fig. 2. Here, the transition is thermodynamically continuous with a RL regime that is of the so-called full replica symmetry breaking (FRSB) kind, associated with a free energy landscape composed by a fractal hierarchy of valleys. As the pumping increases, the regime becomes 1 + FRSB, a combination of 1RSB and FRSB solutions, with both a continuous and a discontinuous contribution to the probability distribution, cf. panel 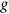 in Fig. 2. The continuous parts in the 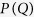 and 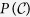 depend on the influence of the off-diagonal damping term in 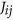 in equation (2). For high enough pumping, well-above the threshold, the non-linear term eventually becomes dominant^15^ and the solution, cf. panel 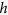 in Fig. 2, eventually becomes 1RSB, as in the closed cavity case, cf. panels *f, g, h* of Fig. 1.

**Figure 3:**
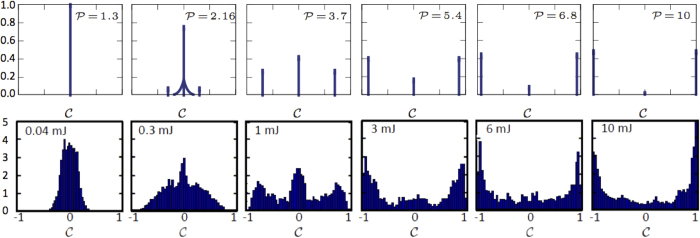
Comparison between theory and experiments in a cavity less random laser. In the top row we display the probability distributions of the IFO for *α* = 0.4, when linear and nonlinear interactions are competing, 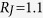 and for increasing pumping. Vertical lines represent Dirac’s deltas, whose height is the probability of the argument value. Different regimes are represented from fluorescence to large pumping random lasing. They are chosen along the dotted line in Fig. 2 at 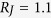. Form left to right the first distribution is at point 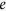 in Fig. 2, the second between 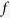 and 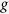, the third one between 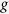 and 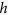 and the following above 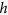. In the bottom row the same regimes are reproduced in the IFO distribution experimentally measured and reported in refs 44,55 in an amorphous solid oligomeric random laser, T5COx.

In the RL experiment of ref. 44 the distribution 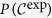, with 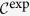 defined in equation (5) is peaked in zero at low pumping, while it becomes nontrivial with a triple and, eventually, double peaked shape as the lasing threshold is overcome. Although in comparison with the theoretical predictions for *N* → ∞ the peaks of 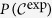 are smeared by noise effects and finite modes’ number effects, in all regimes 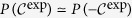. In Fig. 3 we display a comparison between the analytic IFO distribution computed in our 2 + 4 complex amplitude spin-glass model, cf. equation (2), in an open cavity and the experimental measurements of 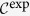 in refs 44,55.

## Discussion

In this work we provide the theoretical analytical background for a recently introduced order parameter^44^ that allows to probe the phenomenon known as replica symmetry breaking in random lasers by means of experimentally accessible observables. These are shot-to-shot intensity fluctuations and the order parameter is the distribution of the values of the overlap between intensity fluctuations in different shots, as analytically defined in equation (6). Replica symmetry breaking is a known property occurring in mean-field glasses, spin-glasses and hard optimization problems. The parameters of the theory have never been measured, though, in any real system in these fields. In, particular, no measurement of the overlap and its distribution has been provided. The only experimental measure, so far, of a quantity possibly related to the standard RSB overlap been recently carried out on a photonic system. The system is an amplifying and scattering random medium, the T5COx^44^ displaying random lasing at high pumping. The parameter is the distribution of the shot-to-shot intensity fluctuations overlap (IFO). In the framework of a recently introduced general statistical mechanics theory of random photonic systems^4^, in equation (21) we give here an analytic proof of the relationship between the IFO and the standard overlap, equation (4), and we provide measurable predictions for its behavior in both ordered and random lasing systems below and above threshold and, furthermore, both in the cases of discontinuous and continuous transitions to the laser regime at the threshold. In particular, the transition in the probability IFO distribution of a random laser is shown to be discontinuous (cf. Fig. 1) for closed (or controllable, limited open) cavities while it becomes continuous (cf. Fig. 2) for highly open cavity nonlinear wave systems. In the cavity-less case, where experimental measurements are available in at least one case, in Fig. 3 we compare theoretical and experimental behavior of the distributions of the IFO 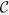 from low to high pumping.

According to our results, a RSB is to be expected only, *though not always*, in random lasers whose random configurations of scatterers are fixed, i.e. *quenched*, for all analyzed shots. That is, the dynamics of their positions evolves on time-scales much longer than the whole experiment and real replicas can be realized. This is the experimental case of the solid/powder samples of random lasers as GaAs powders^54^,^56^, core-shell colloidal CdSe/ZnS quantum dots^57^, ZnO powders^58^ or pressurized pellets^25^, and polymeric substances^44^,^59^,^60^.

Displaying fixed scatterers to realize real replicas is not sufficient to yield a glassy random laser, though. Indeed, as we previously discussed in the Results section, frustration is also necessary. A notable example of a frustration-less solid random laser might be porous gallium phosphide (GaP) filled with a solution of Rhodamine and methanol^61^,^62^, in which spectral fluctuations are reported to be minimal and the structure of the resonances, though random, appears to be reproducible from shot to shot. IFO measurements might yield, in this case, an ordered-like 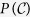, peaked in zero both below and above threshold, as in the phase reported as SML in the diagram of Fig. 2 for 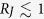.

On the other hand, experiments on optically active random media whose scatterer particles sensitively move between subsequent shots in a single experiment, as in liquid solutions of Rhodamine and methanol with particles of Titanium oxide^63^, Zinc oxide^64^, pure Titania^65^, or colloidal CdSe quantum dots^66^ could establish no real replicas. Not having the same quenched disorder in all shots might prevent the observation of RSB. The overlap between copies of systems with different realizations of the disordered couplings, indeed, is known to be replica symmetric, as it has been shown in models with continuous spherical variables^67^, of which our model in equation (2) is a generalization. Similarly to what happens in the ordered ML case, cf. left panels of Figs 1 and 2, in that case the occurrence of a trivial single peaked 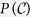 in 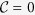 is expected, both below and above 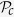. Such a behavior has been observed in a liquid system of TiO_2_ scattering nano-particle suspensions in solution of Rhodamine and methanol^44^.

Eventually, we would like to stress that, besides a rigorous interpretation of recent experimental results for random lasers in terms of replica theory, our results provide an exciting and easily available test of spin-glass theory properties in continuous systems without local magnitude constraints, as disordered photonic systems.

## Methods

### Replica Theory and Order Parameters

The most complicated system that we are considering in our theory is a random system with disordered mode couplings that possibly display a high pumping/low temperature phase with ergodicity breaking and the occurrence of very many states. By “very many” we mean that their number scales with the size of the system, i.e. the number 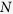 of optically active modes. These states are not related by any simple relationship among them. That is, e.g., no simple 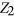 spin reversal symmetry occurs between states, as in the Ising model, nor 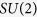 symmetry as in the XY model. In the complex glassy case, to probe the multi-state disordered thermodynamic phase, one, thus, considers 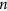 copies of the system with exactly the same set of disordered couplings, the 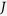
’s, and evaluates the disorder averaged partition function 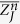 of the replicated system. A continuation to real 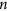 is, then, taken to evaluate


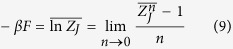


As a result, *F* is expressed as a functional in the replica space of the overlap matrices


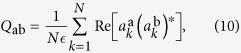



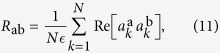



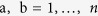
 being replica indexes. The diagonal parts are


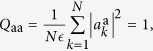


by definition of the total power constraint, and


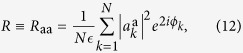


yielding information about global phase coherence. This parameter discriminates between the IW (*R* = 0) and the PLW (*R* > 0) regimes (cf. Figs 1 and 2), in which all the other parameters are identical^14^.

Alternatively, writing 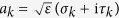, we can define the overlaps of the real parts σ or the imaginary parts *τ* of the complex amplitudes:


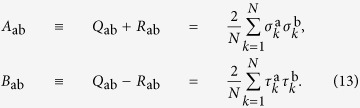


As the system size becomes sufficiently large, the free energy sample-to-sample fluctuations die out and the free energy, equation (9), becomes independent of disorder, i.e., it is *self- averaging*. For *N* → ∞ the physical value of the matrices follows from the extremization of the free energy functional. Because of the fact that the number of independent elements of an overlap matrix is 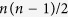 (taken away the diagonal) in the limit *n* → 0 the usual minimization of the thermodynamic potential actually becomes a maximization in the space of the overlap matrices. To maximize *F*, a non-trivial Ansatz on the structure of *Q* and *R* is necessary. Indeed, it can be shown^14^ that the most intuitive *replica symmetric* solution, with *Q*_ab_ and *R*_ab_ independent of a and b, does not lead to a thermodynamically stable solution in the whole phase space: beyond the critical point, in the glassy phase, one must, hence, resort to spontaneous RSB. Following the Parisi scheme^27^ the overlap matrices are, then, taken 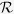-step RSB matrix, with 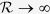 for a continuous full RSB (FRSB). These are block matrices where the number of inner blocks 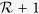 corresponds to the number of hierarchical levels in the multi-state phase space.

Depending on the value of *J*_2,4_ the solution of the RL model Eq. (2) displays phases with different RSB structures, ranging from 1RSB, FRSB to a combination of discontinuous one step and continuous breaking (1 + FRSB)^68^.

### Replicated Action

In the replica formalism, the averages of an observable 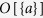 over the equilibrium Gibbs distribution and over the quenched disorder can be written as


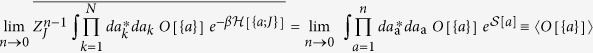


where the average 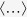 *in the replica space* is evaluated with the replicated action


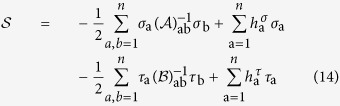


Here we have introduced the matrices


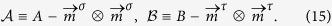


and the effective fields


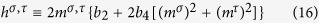


These are functions of the *global coherence* parameters


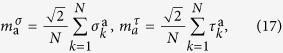


analogous to the magnetization for spin models, with coefficients 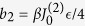, 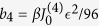. After some algebra (see ref. 15 for details), the field 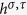 can be expressed as


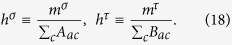


For weak disorder (low 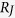) the global coherence 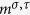 is non-zero above the lasing threshold and must be included into the description. If disorder is strong, though, in the frozen glassy phase, the global coherence is null: 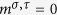.

Because it turns out that 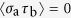 14,15, the integrals in the *σ, τ* space factorize and the IFO 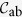 defined in equation (6) takes the form


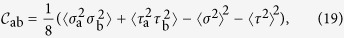


where 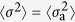 and 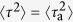, since single replica quantities do not depend on the replica index.

The replicated action 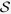 given in equation (14) is quadratic. Thus, using the Wick’s theorem, for the averages in equation (19) we easily obtain


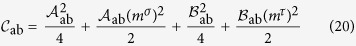


Equation (20) can be further simplified since the physical solutions of the model are either of the form 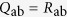 or 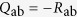 (*a* ≠ *b*). Since the two solutions are equivalent, without loss of generality we choose the first one, so that 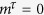 and equation (20) leads to


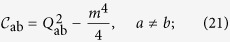



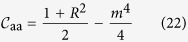


where 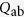 is defined in equation (4),


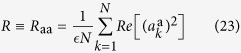


is the parameter of partial coherence, cf. equation (12), and


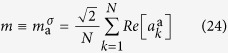


is the parameter of global coherence^14^,^15^. Equation (21) is one of our main results and is discussed in the main text, cf. equation (7).

## Additional Information

**How to cite this article**: Antenucci, F. *et al.* The glassy random laser: replica symmetry breaking in the intensity fluctuations of emission spectra. *Sci. Rep.* **5**, 16792; doi: 10.1038/srep16792 (2015).
